# Protective effects of tea extracts against alcoholic fatty liver disease in mice via modulating cytochrome P450 2E1 expression and ameliorating oxidative damage

**DOI:** 10.1002/fsn3.2526

**Published:** 2021-08-26

**Authors:** Bang‐Yan Li, Qian‐Qian Mao, Ren‐You Gan, Shi‐Yu Cao, Xiao‐Yu Xu, Min Luo, Hang‐Yu Li, Hua‐Bin Li

**Affiliations:** ^1^ Guangdong Provincial Key Laboratory of Food, Nutrition and Health Department of Nutrition School of Public Health Sun Yat‐Sen University Guangzhou China; ^2^ Research Center for Plants and Human Health Institute of Urban Agriculture Chinese Academy of Agricultural Sciences Chengdu China

**Keywords:** alcohol metabolism, alcoholic fatty liver disease, antioxidant activity, lipid accumulation, tea

## Abstract

The alcoholic fatty liver disease (AFLD) has been a severe public health problem. Oxidative stress is involved in the initiation and progression of AFLD. Tea is a popular beverage worldwide with strong antioxidant activity. In this research, our purpose is to explore and compare the effects of 12 selected teas on AFLD. The ethanol liquid diet was used to feed the mice, and 12 tea extracts were administrated at 200 mg/kg body weight every day for 4 weeks. The results showed that the application of several tea extracts exhibited different inhibitory effects on lipid accumulation induced by sub‐acute alcohol consumption based on the determination of triglyceride concentration and the histological alteration in the liver. In addition, several teas significantly decreased serum alanine aminotransferase and aspartate aminotransferase activities, inhibited the cytochrome P450 2E1 expression, and promoted alcohol metabolism (*p* < .05). Besides, compared with the model group, several teas obviously elevated superoxide dismutase and glutathione peroxidase activities as well as glutathione content, and remarkably decreased malondialdehyde level (*p* < .05). In general, Fried Green Tea, Fenghuang Narcissus Oolong Tea, and Pu‐erh Dark Tea possessed potential preventive effects on AFLD. Moreover, the main phytochemicals in the three tea extracts were determined and quantified via high‐performance liquid chromatography, and the most commonly detected ingredients were catechins and caffeine, which could exert the protective effects on AFLD.

## Graphical abstract

Tea is the second most consumed beverage worldwide next to water. Tea contains abundant bioactive ingredients, which exhibit various health benefits, such as antioxidant activity. Tea extracts could effectively protect against fatty liver induced by long‐term and excessive alcohol consumption, might be a potential natural source for preventing alcoholic fatty liver disease.

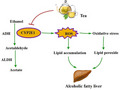

## 1 INTRODUCTION

The alcohol beverage is widely consumed in the world, but alcohol abuse has emerged as serious public health and socioeconomic issue (Llopis et al., 2016; Wang et al., 2014). As revealed by the World Health Organization (WHO), long‐term excessive alcohol consumption (more than 40–80 g/day for males and more than 20–40 g/day for females) was responsible for three million deaths and 132.6 million disability‐adjusted life years (DALYs) in 2016 (Global status report on alcohol and health 2018, 2018). The long‐term excessive alcohol intake can result in alcoholic fatty liver disease (AFLD) which is characterized by abnormal triglyceride accumulation in hepatocytes (Hou et al., 2018). Furthermore, AFLD can progress into alcoholic hepatitis, fibrosis, cirrhosis, and hepatic carcinoma (Lin et al., 2018; Xu et al., 2017). Therefore, prevention of AFLD is very important for the prevention and management of alcohol‐induced liver diseases (Wang et al., 2018). Although the molecular mechanism of the initiation and progression of AFLD is not completely clear, accumulating evidence has suggested that it is related to the activation of cytochrome P450 2E1 (CYP2E1), abnormal lipid metabolism, and oxidative stress (Ceni et al., 2014). Moreover, overwhelming oxidative stress can lead to the generation of lipid peroxide, such as malondialdehyde (MDA), which is an indicator of liver damage (Song et al., 2013). Because oxidative stress plays an essential role in the AFLD, natural products with strong antioxidant properties are attracting substantial attention to prevent and manage AFLD (Hsu et al., 2018).

Tea is a popular beverage worldwide (Shen et al., 2020). Based on the different production processes, tea can be divided into six species, including post‐fermented dark tea, deep‐fermented black tea, semi‐fermented oolong tea, mild‐fermented white tea, slightly fermented yellow tea, and unfermented green tea (Zhao et al., 2014). Tea contains abundant bioactive ingredients, which exhibit various health benefits, such as antioxidant, anticancer, anti‐diabetic activity, anti‐obesity, cardiovascular‐protective, and hepatoprotective effects (Cao et al., 2019; Meng et al., 2018). In this study, a mouse model of alcoholic fatty liver was used to investigate and compare the effects of 12 selected teas on AFLD. The research results can provide references for the public to choose tea for the prevention of AFLD. In addition, several teas can be also developed into functional food to prevent and manage AFLD.

## 2 EXPERIMENTAL MATERIALS AND METHODS

### 2.1 Experimental reagents

The 95% ethanol was bought from Guangzhou Wego Instrument Co., Ltd. The detection kits of aspartate transaminase (AST), serum alanine transaminase (ALT), triglyceride (TG), and total cholesterol (TC) in serum were purchased from Roche diagnostics (Shanghai, China). In addition, the kits of alcohol dehydrogenase (ADH), acetaldehyde dehydrogenase (ALDH), superoxide dismutase (SOD), catalase (CAT), glutathione peroxidase (GSH‐Px), and glutathione (GSH) were obtained from Nanjing Jiancheng Bioengineering Institute. Also, the level of CYP2E1 was assessed using CYP2E1 ELISA Kit (Meimian). Moreover, the determination kits of TG, MDA, and total protein were purchased from Apply‐gen Technologies Inc. Furthermore, the standard chemical compounds used in high‐performance liquid chromatography (HPLC) analysis were provided by Chengdu Derick Biotechnology Co., Ltd., including gallic acid, gallocatechin (GC), epigallocatechin (EGC), catechin (C), chlorogenic acid, caffeine, epigallocatechin gallate (EGCG), epicatechin (EC), gallocatechin gallate (GCG), epicatechin gallate (ECG), catechin gallate (CG), ellagic acid, astragalin, myricetin, quercitrin, quercetin, theaflavin, and kaempferol. All other chemical reagents were of analytical grade.

### 2.2 Preparation of tea extracts and alcohol‐containing liquid diet

The 12 teas were purchased from China. Table 1 has shown the specific information about the 12 selected teas. The tea samples (10 g) were extracted with 100 ml of boiling deionized water in a 98°C water bath shaker‐DKZ‐450B (Sensin) for 10 min, and the extraction steps were repeated three times (Cao et al., 2020). All extracted solutions were combined and filtered. Subsequently, the extracted solutions were concentrated to about 15 ml by a vacuum rotary evaporator (R‐501, Cancun, Shanghai, China). Furthermore, the concentrated extracted solutions were freeze‐dried into powders in a freeze drier (Labconco‐7752001). The dried tea extracts were stored at −80°C for further experiments. The powders of tea extracts were dissolved in distilled water at 20 g/L (w/v) for administration to the mice.

**TABLE 1:** The information of 12 selected teas

No.	Name	Category	Fermentation degree	Production place
GT1	Selenium‐Enriched Enshi Yulu Tea	Green Tea	Unfermented	Enshi, Hubei
GT2	Fried Green Tea	Green Tea	Unfermented	Shaoxing, Zhejiang
YT1	Yuan'an Luyuan Tea	Yellow Tea	Light‐fermented	Yichang, Hubei
YT2	Mengding Huangya Tea	Yellow Tea	Light‐fermented	Mengdingshan, Sichuan
WT1	Gongmei White Tea	White Tea	Mild‐fermented	Fuzhou, Fujian
WT2	White Peony Tea	White Tea	Mild‐fermented	Fuzhou, Fujian
OT1	Wuyi Narcissus Tea	Oolong Tea	Semi‐fermented	Wuyishan, Fujian
OT2	Fenghuang Narcissus Tea	Oolong Tea	Semi‐fermented	Shantou, Guangdong
BT1	Yihong Tea	Black Tea	Deep‐fermented	Yichang, Hubei
BT2	Lapsang Souchong Tea	Black Tea	Deep‐fermented	Xiamen, Fujian
DT1	Qing Brick Tea	Dark Tea	Post‐fermented	Yichang, Hubei
DT2	Pu‐erh Tea	Dark Tea	Post‐fermented	Pu'er, Yunnan

The alcoholic fatty liver was induced by Lieber‐DeCarli ethanol liquid diet (TROPHIC Animal Feed High‐tech Co., Ltd.), with an energy composition of carbohydrate, protein, and fat of 19%, 18%, and 35%, respectively, while ethanol containing in the liquid diet supplied 28% of total calories. In addition, animals in the control group were fed with a Lieber‐DeCarli control liquid diet (TROPHIC Animal Feed High‐tech Co., Ltd., Nantong, China), in which ethanol was substituted by carbohydrates. According to the manufacture's instruction, all the liquid diets were made from powders every day. For alcohol‐containing liquid diet (1 Kcal/ml), 0.53 g of choline, 2.5 g of vitamins, and 53.3 ml of 95% ethanol (4%, w/v) were blended to 147.0 g liquid diet powder and mixed with 40°C distilled water to make up to 1000.0 ml. Table 2 has listed the ingredients, and calories contained in a liter of liquid diet.

**TABLE 2:** The composition and calories of liquid diet (one‐liter liquid diet)

Diet calories and composition	Control liquid diet	Alcohol‐containing liquid diet
Diet calories		
Carbohydrate (%)	47.0	19.0
Protein (%)	18.0	18.0
Fat (%)	35.0	35.0
Ethanol (%)	—	28.0
Total (%)	100.0	100.0
1 ml liquid diet (Kcal)	1.0	1.0
Diet compositions		
Liquid diet powder (g)	218.8	147.0
Choline (g)	0.53	0.53
Vitamins (g)	2.5	2.5
95% Ethanol (ml)	—	53.3
Water (mL, 40°C )	Volume to 1000.0	Volume to 1000.0

### 2.3 Animal and experimental design

**FIGURE 1:**
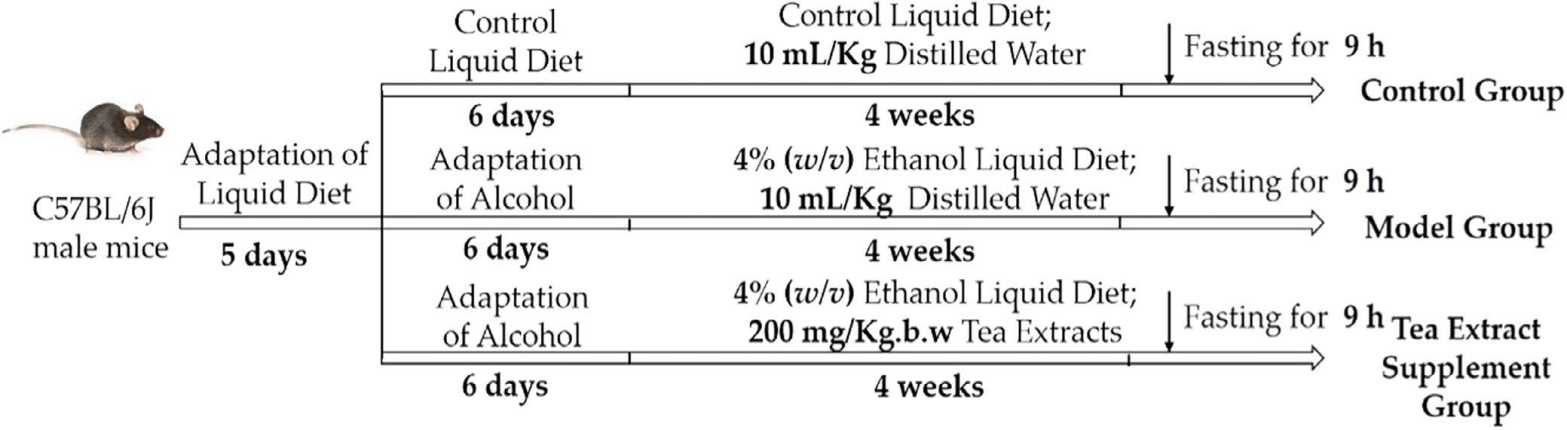
The animal experimental progress of the effect of tea extracts on AFLD. During the experimental period, the liquid diets were freshly prepared, and replaced at 6:00 o'clock (p.m.) every day. Moreover, the body weight of mice was weighed two times a week. The daily intake of liquid diets was recorded, and the feeding bottles were autoclaved (120°C, 30 min) every day

C57BL/6J male mice (20 g) were obtained from the Experimental Animal Center of Guangdong province, Guangzhou, China. The animal experiment was conducted in accordance with the "Principles of Laboratory Animal Care and Use” approved by the School of Public Health, Sun Yat‐Sen University (No. 2019‐002; 28 February 2019). Mice were housed in an animal room of specific pathogen‐free (SPF). The light/dark cycle of the animal room is 12 hr. The temperature and relative humidity are 22 ± 0.5°C and 40%–60%, respectively. Because of the particularity of liquid diet feed, the mice were divided into small cages with three mice per cage. After acclimatizing to the environment, the mice were fed Lieber‐DeCarli control liquid diet feed for 5 days. Subsequently, the mice were randomly divided into different groups (9 mice in each group) in the light of body weight, including control (CTRL), model (EtOH), and tea extract treatment (EtOH+Tea) groups. From the sixth day, the model group and tea extract treatment groups were performed alcohol adaptation for 6 days, which were gradually fed with the mixture of Lieber‐DeCarli ethanol and control liquid diet at the ratio of 1:2, 1:1, and 2:1. After the alcohol adaptation, the model group and tea extract treatment groups were fed with the Lieber‐DeCarli ethanol liquid diet, while the control group mice were pair‐fed as previously described. On the 12th day, mice in the tea extract treatment groups were fed intragastrically with tea extracts at the dose of 200 mg/kg.b.w for 4 weeks according to the reports, which have been demonstrated that the dose range of 100–400 mg/kg.b.w tea extracts can play strong protective effects on alcohol‐induced liver damage (Martins et al., 2017; Seo et al., 2017). At the same time, the control and model groups were treated with distilled water (10 ml/kg) for 4 weeks. At the end of the experiment, all the mice fasting for 9 hr were weighed and deeply anesthetized to sacrifice. Moreover, the blood and liver samples were collected and used for further experiments. The animal experimental progress is displayed in Figure 1.

### 2.4 Biochemical analysis of serum

The blood samples were immediately collected by removing the eyeball and centrifuged at 4000 × *g* for 10 min to collect the serum after it was kept at room temperature for one hour. The serum was isolated for AST, ALT, TG, and TC evaluation, which were measured by the automated biochemistry analyzer (Roche, Mannheim, Germany) and according to the manufacturer's instructions. Commercial enzymatic tests were performed for TG and TC determination, while velocity tests were used for AST and ALT detection.

### 2.5 Biochemical analysis of liver sample

After the mouse liver was separated, it was washed with ice‐cold 0.9% saline solution, and then sucked by the filter paper and weighed. A part of the right lobe of the liver sample was fixed with 4% (w/v) paraformaldehyde, and the rest was stored at −80°C for biochemical assay.

#### 2.5.1 Determination of hepatic TG contents

Liver samples were washed two times with phosphate‐buffered saline (PBS) before analysis. The liver homogenate was made by mixing the frozen liver and lysis buffer (20 mg/ml) and incubated at 4°C for 30 min, and then was ground with TissueLyser II Qiagen (QIAGEN^®^). The liver homogenate was heated at 70°C for 10 min and centrifuged at 4°C for 5 min at 2000 × *g* to obtain the supernatant. The TG concentration in the supernatant was determined with TG commercial kits by enzymatic colorimetric assay. In addition, a part of the liver homogenate was used to measure total protein content with BCA commercial kit to correct the contents of TG. The liver homogenate to 0.25% (w/v) was diluted with lysis buffer and then mixed 10 µl of liver homogenate with the working reagent. The mixture was placed in a 37°C water bath for 15 min, and the absorbance of the mixture was measured at 550 nm using a microplate reader.

#### 2.5.2 Determination of ADH and ALDH activities as well as CYP2E1 content

Hepatic ADH activity was evaluated in accordance with the instruction of the kit, which is based on the principle of ADH catalyzing the reaction of oxidized nicotinamide adenine dinucleotide (NAD^+^) to reduced nicotinamide adenine dinucleotide (NADH). Briefly, the liver tissues from centrilobular of mouse liver were homogenized at 1:9 ratio (w/v) in ice‐cold 0.9% normal saline solution with a glass grinder. The 10% liver homogenate was centrifuged at 2500 × *g* for 10 min at 4°C to obtain the supernatant, and then 0.05 ml supernatant was added to the prepared mixture to initiate the reaction in a 37°C water bath. The absorbance of the mixture was immediately detected at 340 nm after 15 s and was determined again after 10 min.

Hepatic ALDH activity was determined using the commercial kit according to the theory that acetaldehyde, and NAD^+^ can be converted into acetic acid and NADH under the catalysis of ALDH and the absorbance increases at 340 nm. The 10% liver homogenate was prepared as described in the detection of ADH activity in this study. After centrifugation for 20 min at 10,000  × g and 4°C, 0.2 ml supernatant was mixed with the prepared reagents and put in a water bath at 37°C. The absorbance of the mixture was immediately measured at 340 nm after 10 s and was detected again after 1 min.

The level of CYP2E1 was monitored by using the double antibody sandwich method with the CYP2E1 ELISA kit. The 10% liver homogenate was made by mixing the frozen liver (20 mg) and PBS (180 µl) and was centrifuged at 3000 × *g* for 20 min at 4°C to receive the supernatant. Moreover, the supernatant was diluted by PBS to 2% (w/v), which was used for the determination of the CYP2E1 level based on the instructions of CYP2E1 ELISA kit. In brief, 10 µl mouse CYP2E1 antibody was added to the microtiter plate wells, and then 40 µl supernatant was added. The CYP2E1 in the supernatant combines with CYP2E1 antibody that is labeled with the horseradish peroxidase (HRP) to form antibody‐antigen‐enzyme‐antibody complex. The 100 µl 3,3′,5,5′‐tetramethylbenzidine (TMB) substrate solution was added after washing completely. TMB substrate becomes blue color by the enzyme‐catalysis of HRP and is converted to yellow color after the addition of a 50 µl sulfuric acid solution. The absorbance was detected at 450 nm by the microplate reader.

#### 2.5.3 Determination of antioxidant activity and MDA level

The liver homogenate was made by mixing the liver tissue and ice‐cold normal saline solution (0.9%) in a glass homogenate tube; then, the homogenate was centrifuged at 2500 × *g* for 10 min at 4°C to gain the supernatant for the determination of antioxidant activity. The procedure used for the determination of CAT, SOD, and GSH‐Px activities, and the GSH content was performed based on the manufacturer's protocols. In addition, a part of the liver homogenate was used to measure total protein content with BCA commercial kit to correct the activities of SOD, CAT, and GSH‐Px and the content of GSH as described previously.

The hepatic MDA content was assessed according to the instruction of the Apply‐gen commercial kit. MDA and thiobarbituric acid (TBA) were reacted at a higher temperature and acidic environment to form red MDA‐TBA adduct, which has the maximum absorption at 535 nm. Liver tissue was washed with 10% PBS containing 1 mM ethylene diamine tetraacetic acid (EDTA) to remove blood, and the liver homogenate was made as described in the determination of hepatic TG content in this study. The mixture of 100 µl liver homogenate and the reagent (300 µl) was heated at 95°C for 30 min and centrifuged for 10 min at 10000 × *g* at 4°C to gain the supernatant. Eventually, 200 µl supernatant was measured at 535 nm to obtain the absorbance.

### 2.6 Histopathological analysis of liver

Liver samples were fixed with 4% (w/v) paraformaldehyde and left at room temperature for 2 days. The liver histological evaluation was performed by two methods, including hematoxylin–eosin (H&E) staining and Oil Red O staining. The paraffin‐embedded liver slices were stained with hematoxylin–eosin (H&E) staining. Oil Red O staining was performed on liver frozen sections. The images were captured with a light microscope (Leica).

### 2.7 Measurement of phytochemicals in teas

The phytochemical components in tea extracts were detected by High‐Performance Liquid Chromatography (HPLC) based on a previous report (Cai et al., 2018), which was consisted of a photodiode array detector (Waters 2996, USA), an Agilent Zorbax Eclipse XDB‐C18 column (4.6 × 250 mm, 5 µm, Santa Clara, CA, USA) and an HPLC pump separation module (Waters 1525, Milford, MA, USA). The mobile phase A and B were 0.1% formic (v/v) and methanol, respectively. In addition, the temperature of separation was 35°C and the flow rate was 1.0 ml/min. Moreover, the elution gradient performed as shown in Table 3. Furthermore, phytochemicals were identified based on the retention time and the UV‐Vis spectrum of the standard, and quantified according to the peak area at the maximum absorption wavelength.

**TABLE 3:** Mobile phase gradient elution program

Time/min	Methanol%
0	2
10	17
15	19
20	22
40	47
50	50
60	58
70	2
75	2

### 2.8 Statistical analysis

The experimental results were expressed as mean ± standard deviation (*SD*). Comparisons between groups were analyzed by one‐way analysis of variance (ANOVA) and Dunnett‐*t* test. Statistical analysis was performed using SPSS software 20.0 (IBM SPSS Statistics, IBM Corp), and a statistical difference with *p* < .05 was significant. The GraphPad Prism 8 software and excel were used to draw the graph.

## 3 RESULTS AND DISCUSSION

### 3.1 Effects of tea extracts on body weight

The effects of 12 tea extracts on body weight of alcohol‐induced mice are shown in Table 4. The model group showed a non‐significant decrease in body weight compared with the control group. There was no significant change in body weight in most tea extract treatment groups in comparison with the model group. However, the body weight in the Mengding Huangya Tea (YT2) group was lower than that in the model group in the first to fourth weeks (*p* < .05). Perhaps, this tea contained some substances that could inhibit weight gain, and need to be further studied in the future.

**TABLE 4:** The effects of 12 teas on body weight of mice (g)

Groups	After adaptation of liquid diet	After adaptation of alcohol	First week	Second week	Third week	Fourth week
CTRL	23.54 ± 1.79	23.85 ± 1.92	24.05 ± 1.69	25.42 ± 1.88	26.07 ± 1.99	26.63 ± 1.39
EtOH	23.64 ± 1.53	23.55 ± 1.77	23.62 ± 1.68	24.32 ± 2.07	25.13 ± 1.93	25.90 ± 2.13
GT1	24.58 ± 1.21	25.06 ± 1.63	24.62 ± 1.91	25.54 ± 2.08	26.12 ± 2.06	26.59 ± 1.84
GT2	23.43 ± 1.45	23.69 ± 1.44	23.53 ± 1.50	24.51 ± 1.66	24.58 ± 1.86	25.53 ± 1.54
YT1	24.44 ± 1.37	24.34 ± 1.09	24.17 ± 1.27	24.53 ± 1.46	24.96 ± 1.69	25.66 ± 1.32
YT2	23.00 ± 1.22	22.81 ± 1.28	22.07 ± 1.78*	22.84 ± 1.78*	23.16 ± 1.40*	23.66 ± 1.32*
WT1	23.70 ± 0.39	23.70 ± 0.50	23.84 ± 0.78	24.31 ± 1.29	24.75 ± 1.82	25.35 ± 1.99
WT2	23.83 ± 1.13	24.32 ± 0.89	24.31 ± 0.66	25.09 ± 0.77	25.27 ± 0.98	25.84 ± 0.97
OT1	24.22 ± 0.73	24.36 ± 0.71	24.77 ± 0.96	24.11 ± 2.08	25.68 ± 1.23	26.23 ± 0.97
OT2	24.14 ± 1.97	24.40 ± 1.83	23.81 ± 1.80	24.53 ± 1.15	24.62 ± 1.79	25.23 ± 1.53
BT1	23.94 ± 1.60	24.10 ± 1.57	24.26 ± 1.39	24.52 ± 1.20	25.08 ± 1.37	25.08 ± 1.45
BT2	24.39 ± 1.13	24.18 ± 1.13	24.08 ± 1.04	24.32 ± 1.61	25.03 ± 1.93	26.29 ± 1.57
DT1	24.24 ± 1.77	24.33 ± 1.70	24.01 ± 2.02	24.31 ± 1.64	24.93 ± 1.78	25.35 ± 1.69
DT2	24.61 ± 1.02	24.83 ± 1.80	24.44 ± 1.82	24.97 ± 1.67	25.61 ± 1.48	26.61 ± 1.33

Note

Values are expressed as mean ± *SD*.

Abbreviations: BT1, Yihong Tea; BT2, Lapsang Souchong Tea; CTRL, the control group; DT1, Qing Brick Tea; DT2, Pu‐erh Tea. EtOH, the model group; GT1, Selenium‐Enriched Enshi Yulu Tea; GT2, Fried Green Tea; OT1, Wuyi Narcissus Tea; OT2, Fenghuang Narcissus Tea; WT1, Gongmei White Tea; WT2, White Peony Tea; YT1, Yuan'an Luyuan Tea; YT2, Mengding Huangya Tea.

*p <.05, the tea extract treatment group versus the model group.

In general, chronic alcohol consumption and alcohol metabolism could influence body weight due to the reduction of feed intake caused by the stimulation of alcohol and the energy wastage induced by alcohol metabolism (Hsu et al., 2018; Pirola & Lieber, 1976). For example, a study reported that the body weight of the model group obviously decreased compared with the control group (Hsu et al., 2018). However, the results from this study showed that there was no significant difference in body weight among the control group, model group, and most tea extract treatment groups during the experimental period. The possible reason was the liquid diets in the control and tea extract treatment groups in this study were given according to the model group's feed intake the day before (pair‐fed), which could ensure an equal energy intake, but all mice had free access to liquid diets in previous study (Hsu et al., 2018).

### 3.2 Effects of tea extracts on serum AST and ALT

**FIGURE 2:**
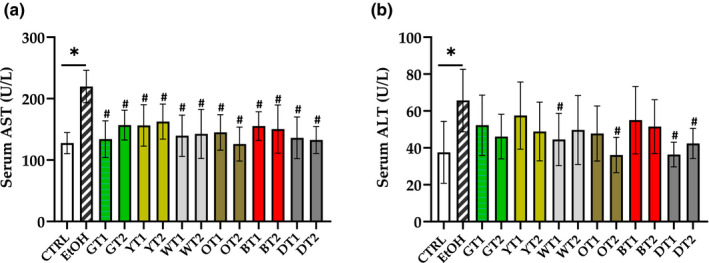
The effects of 12 teas on liver injury in mice with fatty liver induced by sub‐acute alcohol consumption. (a) AST, aspartate transaminase; (b) ALT, alanine aminotransferase. CTRL, the control group; EtOH, the model group; GT1, Selenium‐Enriched Enshi Yulu Tea; GT2, Fried Green Tea; YT1, Yuan'an Luyuan Tea; YT2, Mengding Huangya Tea; WT1, Gongmei White Tea; WT2, White Peony Tea; OT1, Wuyi Narcissus Tea; OT2, Fenghuang Narcissus Tea; BT1, Yihong Tea; BT2, Lapsang Souchong Tea; DT1, Qing Brick Tea; DT2, Pu‐erh Tea. *p < .05, the model group versus the control group; *#p* <.05, the tea extract treatment group versus the model group

The effects of 12 teas against AFLD were evaluated by determining the levels of AST and ALT. As displayed in Figure 2, compared with the control group, mice in the model group showed a significant increase in the levels of AST and ALT (*p* < .05), suggesting the presence of liver damage.

Seen from Figure 2a, the level of serum AST was notably decreased in all treatment groups in comparison with the model group (*p* < .05), but there was no significant difference among treatment groups. In Figure 2b, Fenghuang Narcissus Tea (OT2) showed the strongest ALT‐reducing activity among the four tea extracts that could reduce the level of serum ALT (*p* < .05). The combination of the results from AST and ALT showed that Gongmei White Tea (WT1), Fenghuang Narcissus Tea (OT2), Qing Brick Tea (DT1), and Pu‐erh Tea (DT2) could significantly decrease the levels of both AST and ALT in serum. It was suggested that these teas showed protective effects against liver damage induced by alcohol. However, Selenium‐Enriched Enshi Yulu Tea (GT1), Fried Green Tea (GT2), Yuan'an Luyuan Tea (YT1), Mengding Huangya Tea (YT2), White Peony Tea (WT2), Wuyi Narcissus Tea (OT1), Yihong Tea (BT1), and Lapsang Souchong Tea (BT2) only reduced the level of AST (*p* < .05). This could be because their concentrations are too small since other conditions (such as treatment duration and administration method) of 12 teas were the same in this study. For example, a previous study reported that only the level of AST could be reduced in the intervention of 1% aqueous extract of pepino (*Solanum muriactum* Ait) leaves (AEPL) in AFLD mice model, but 2% AEPL could decrease the levels of AST and ALT (Hsu et al., 2018).

### 3.3 Effects of tea extracts on TG and TC, and liver coefficient

**FIGURE 3:**
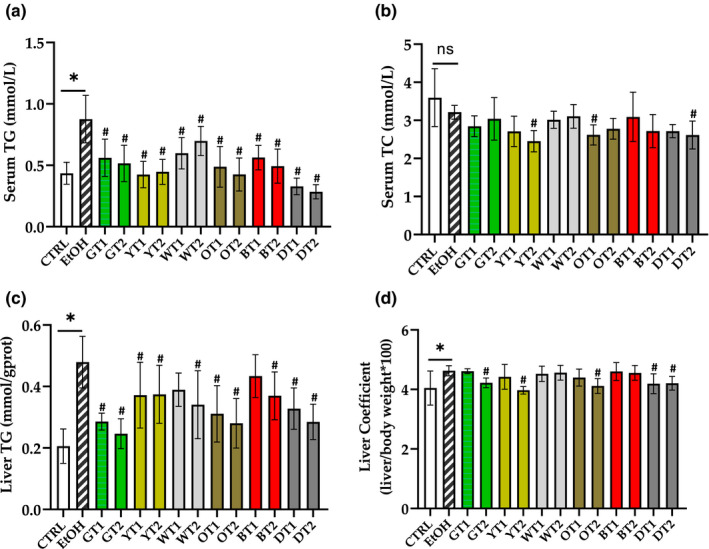
The effects of 12 teas on lipid metabolism in mice with fatty liver induced by sub‐acute alcohol consumption. (a) Serum TG, serum triacylglycerol; (b) Serum TC, Serum total cholesterol; (c) Liver TG, Liver triacylglycerol; (d) Liver Coefficient. CTRL, the control group; EtOH, the model group; GT1, Selenium‐Enriched Enshi Yulu Tea; GT2, Fried Green Tea; YT1, Yuan'an Luyuan Tea; YT2, Mengding Huangya Tea; WT1, Gongmei White Tea; WT2, White Peony Tea; OT1, Wuyi Narcissus Tea; OT2, Fenghuang Narcissus Tea; BT1, Yihong Tea; BT2, Lapsang Souchong Tea; DT1, Qing Brick Tea; DT2, Pu‐erh Tea. *p < .05, the model group versus the control group; #*p* < .05, the tea extract treatment group versus the model group

As depicted in Figure 3, the model group showed an obvious increase (*p* < .05) in the concentrations of TG in serum and liver as well as the liver coefficient. However, there was a non‐significant difference in the content of TC in serum when compared with the control group. In addition, seen from Figure 3a,c, most of the 12 tea extracts markedly reduced (*p* < .05) the level of TG in serum and liver compared with the model group. Moreover, the effect of 12 tea extracts on the liver coefficient is presented in Figure 3d. The elevation in the liver coefficient was reversed (*p* < .05) in five out of 12 treatment groups compared with the model group. On the other hand, Figure 3b illustrates the effects of 12 tea extracts on TC content in serum. Compared with the model group, the serum TC concentration decreased (*p* < .05) in some treatment groups.

According to the results, sub‐acute consumption of alcohol resulted in a significant elevation in the concentrations of TG in serum and liver. In addition, most of the 12 teas obviously decreased serum and hepatic TG concentrations, which indicated that they could prevent abnormal TG accumulation. This might be because these teas reduced the production of ROS by improving alcohol metabolism and elevating antioxidant activity (Fischer et al., 2003). Moreover, Fried Green Tea (GT2), Fenghuang Narcissus Tea (OT2), and Pu‐erh Tea (DT2) showed stronger inhibitory effects on liver lipid accumulation, while Selenium‐Enriched Enshi Yulu Tea (GT1) and black teas had the weakest effects, which was in agreement with the previous result in this research, where these teas did not significantly decrease the level of ALT.

In this study, we found that there were no significant changes in serum TC content between the model group and the control group. A previous study showed an increase in the content of serum TC after 5 weeks of alcohol consumption in the model group (Hsu et al., 2018), while another study revealed that the serum TC concentration did not increase after 9 months, but it elevated after 12 months (Tan et al., 2017). This difference could be related to the different treatment duration, animal species, form of feed, composition of energy in the feed, and so on. On the other hand, Mengding Huangya Tea (YT2), Wuyi Narcissus Tea (OT1), and Pu‐erh Tea (DT2) reduced the content of TC in serum, which showed certain tea extracts might provide a preventive effect on the increase of TC after alcohol consumption.

The sub‐acute intake of alcohol could increase the liver coefficient compared with the control group in the present study, and the result was consistent with a previous study (Wang et al., 2018). Moreover, the liver coefficient was reduced in Fried Green Tea (GT2), Mengding Huangya (YT2), Fenghuang Narcissus Tea (OT2), Qing Brick Tea (DT1), and Pu‐erh Tea (DT2) groups compared with the model group, which further confirmed the preventive effect of these teas on AFLD.

In short, several teas could provide different inhibitory effects on lipid accumulation in the liver after sub‐acute alcohol consumption, especially Fried Green Tea (GT2), Fenghuang Narcissus Tea (OT2), and Pu‐erh Tea (DT2). However, Yihong Tea (BT1) cannot inhibit the hepatic lipid accumulation.

### 3.4 Histopathological evaluation

**FIGURE 4:**
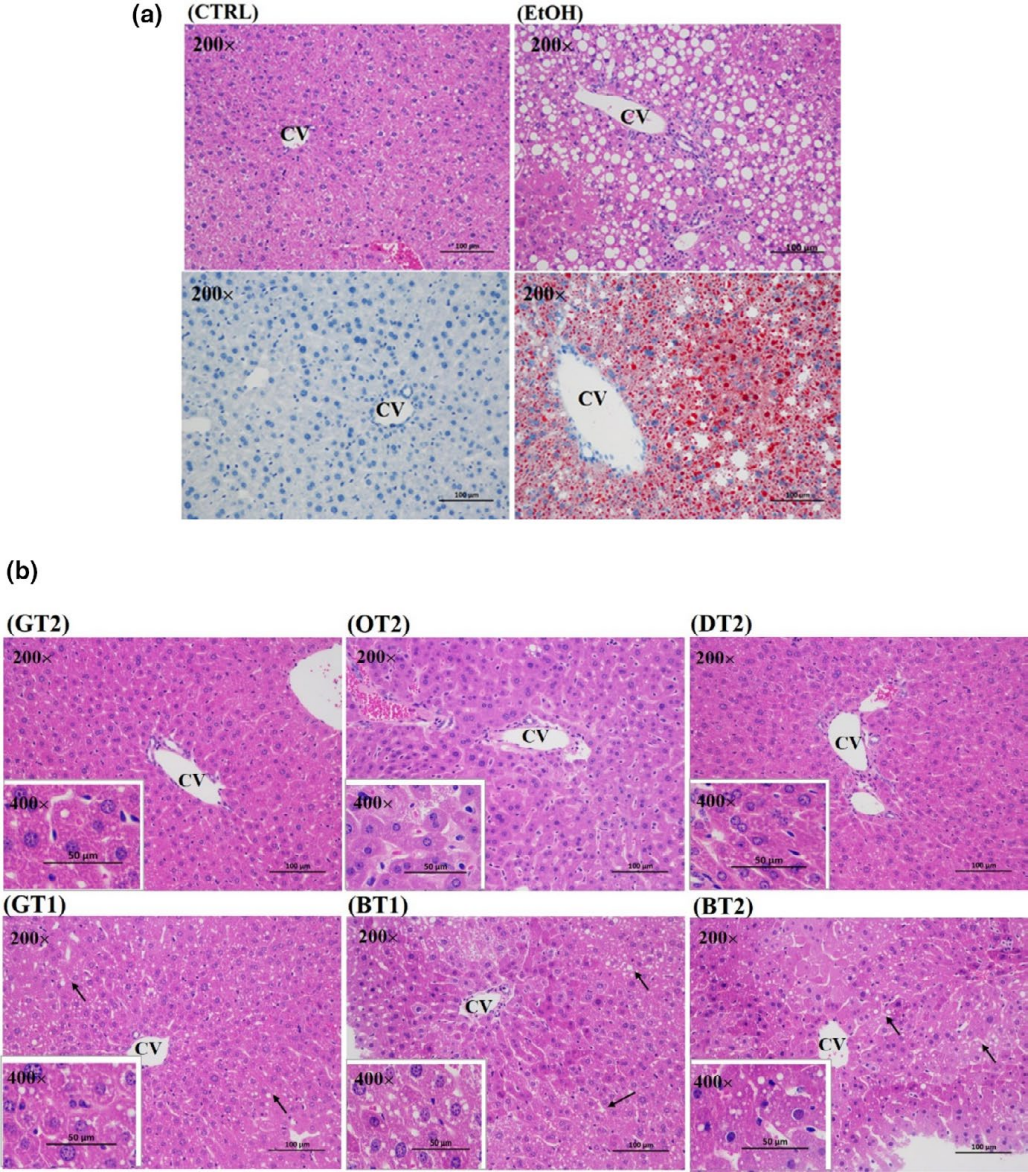
The Morphological examination of hematoxylin and eosin (H&E) stained for all groups and Oil Red O staining for control and model groups (magnification: 200, scale bar: 100 μm; magnification: 400, scale bar: 50 μm). (a) CTRL, the control group; EtOH, the model group; (b) GT2, Fried Green Tea; OT2, Fenghuang Narcissus Tea; DT2, Pu‐erh Tea; GT1, Selenium‐Enriched Enshi Yulu Tea; BT1, Yihong Tea; BT2, Lapsang Souchong Tea

In order to further investigate the inhibitory effects of 12 teas against the hepatic lipid accumulation induced by chronic consumption of alcohol, the histopathological assessment was performed on all groups (Figure 4). As displayed in Figure 4a, hematoxylin–eosin (H&E) staining and Oil Red O staining showed a large amount of tiny to medium lipid droplets in the hepatocytes of the model group, while no obvious pathological abnormality was observed in the control group.

Seen from Figure 4b, the Fried Green Tea (GT2), Fenghuang Narcissus Tea (OT2), and Pu‐erh Tea (DT2) extract treatment groups presented less pathological changes, indicating that they had a stronger preventive effect against fatty liver induced by chronic alcohol intake. However, the liver of mice in the Selenium‐Enriched Enshi Yulu Tea (GT1), Yihong Tea (BT1), and Lapsang Souchong Tea (BT2) groups showed a small number of tiny lipid droplets in hepatocyte cytoplasm compared with the model group. It is suggested that these teas exhibited weaker protective effects against lipid accumulation in hepatocytes induced by sub‐acute alcohol consumption.

### 3.5 Effects of tea extracts on CYP2E1 expression and alcohol metabolism

**FIGURE 5:**
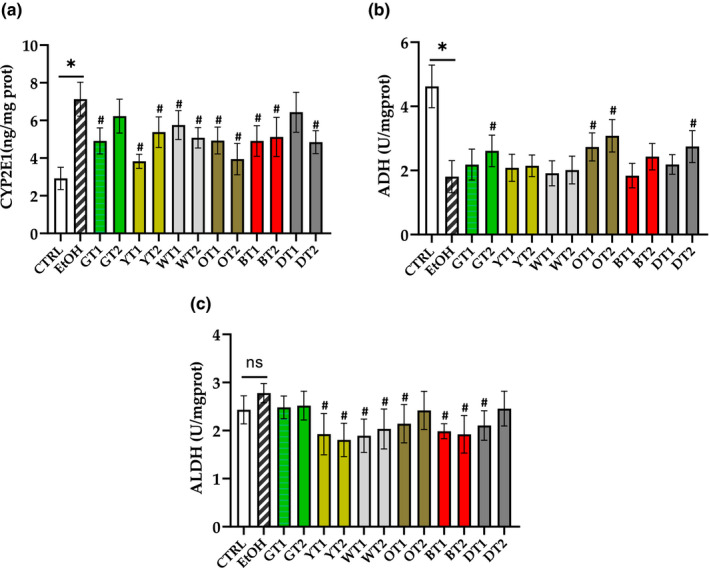
The effects of 12 teas on alcohol metabolism in mice with fatty liver induced by sub‐acute alcohol consumption. (a) CYP2E1, cytochrome P450 2E1; (b) ADH, alcohol dehydrogenase; (c) ALDH, aldehyde dehydrogenase. CTRL, the control group; EtOH, the model group; GT1, Selenium‐Enriched Enshi Yulu Tea; GT2, Fried Green Tea; YT1, Yuan'an Luyuan Tea; YT2, Mengding Huangya Tea; WT1, Gongmei White Tea; WT2, White Peony Tea; OT1, Wuyi Narcissus Tea; OT2, Fenghuang Narcissus Tea; BT1, Yihong Tea; BT2, Lapsang Souchong Tea; DT1, Qing Brick Tea; DT2, Pu‐erh Tea. *p < .05, the model group versus the control group; # *p* < .05, the tea extract treatment group versus the model group

As indicated in Figure 5, the expression of CYP2E1 was remarkably increased (*p* < .05) and the activity of ADH was significantly inhibited (*p* < .05) in the model group compared with the control group. However, there was no significant difference in the activity of ALDH between the model group and the control group. In the literature, it has been reported that more than 90% of ethanol metabolism occurred in the liver (Bourogaa et al., 2013). Generally, ethanol is metabolized through a two‐step process, in which ADH metabolizes ethanol to acetaldehyde, and acetaldehyde is further oxidized to acetic acid by ALDH (Gu et al., 2012). In addition, the speed of the oxidation of ethanol to acetaldehyde and the conversion of acetaldehyde into acetic acid can be increased when the activities of ADH and ALDH are boosted (Zhang, Wang, et al., 2016). Moreover, it has been demonstrated that chronic heavy alcohol consumption induced the activation of CYP2E1 to replace ADH, which can lead to the generation of excessive ROS and acetaldehyde (Caro & Cederbaum, 2004). Based on our results, the expression of CYP2E1 was evidently increased in the model group, indicating that excessive ROS was produced in the liver after sub‐acute excessive alcohol intake, which could contribute to severe oxidative stress damage in hepatocytes.

Seen from Figure 5a, the hepatic CYP2E1 expression was obviously decreased (*p* <.05) in most of the tea extract treatment groups, especially Fried Green Tea (GT2), Yuan'an Luyuan Tea (YT1), Fenghuang Narcissus Tea (OT2), and Pu‐erh Tea (DT2). It is indicated that they could reduce the accumulation of ROS and the level of oxidative stress. The results were agreed with a previous in vitro study which showed an inhibitory effect on overexpressing CYP2E1 in the HepG2 cell model treated with catechin and caffeine ingredients from green tea (Jimenez‐Lopez & Cederbaum, 2004). It has been reported that catechins were the major polyphenols in tea extracts, which could be responsible for the hepatoprotective effect of tea (Zhao et al., 2019). Besides, it has been indicated that the level of polyphenols in tea with higher fermentation levels may be lower, since tea polyphenols (especially catechins) can be oxidized and polymerized during fermentation (Pereira‐Caro et al., 2017). Seen from the results, Qing Brick Tea (DT1) did not remarkably reduce the expression of CYP2E1 in this study, it might be because that post‐fermentation of the dark tea decreased the content of polyphenols. However, the significant decrease of CYP2E1 expression was not observed in the Selenium‐Enriched Enshi Yulu Tea (GT1) which was unfermented. It might be because the content of polyphenols in tea was also affected by other factors like the maturity of tea. A study has reported that polyphenols content in tea was negatively correlated with the maturity of tea leaves (Baptista et al., 2012).

As shown in Figure 5b, the Fried Green Tea (GT2), Wuyi Narcissus Tea (OT1), Fenghuang Narcissus Tea (OT2), and Pu‐erh Tea (DT2) significantly increased (*p* < .05) the activity of ADH compared with the model group. It illustrates that these teas could promote process of alcohol metabolism and elevate the speed of the oxidation of ethanol to acetaldehyde, exerting potential health effects after alcohol consumption. On the other hand, Yuan'an Luyuan Tea (YT1), Mengding Huangya Tea (YT2), Gongmei White Tea (WT1), White Peony Tea (WT2), Wuyi Narcissus Tea (OT1), Yihong Tea (BT1), Lapsang Souchong Tea (BT2), and Qing Brick Tea (DT1) significantly inhibited the activity of ALDH (*p* < .05). The reduction in ALDH activity could decrease the conversion of acetaldehyde into acetic acid and increase the accumulation of acetaldehyde in the body system. Acetaldehyde is more harmful than ethanol and could cause toxic effects, such as lightheadedness, a rapid pulse, sweating, nausea, and vomiting (Zhang, Wang, et al., 2016). The results indicated the side effect of these teas against the intake of alcohol.

Overall, most of the 12 teas could inhibit the expression of CYP2E1 induced by sub‐acute alcohol consumption (Figure 7). Some of the 12 teas could play potential protective effects on alcohol metabolism, especially Selenium‐Enriched Enshi Yulu Tea (GT1), Fried Green Tea (GT2), Fenghuang Narcissus Tea (OT2), and Pu‐erh Tea (DT2).

### 3.6 Effects of tea extracts on antioxidant capacity and lipid peroxidation level

**FIGURE 6:**
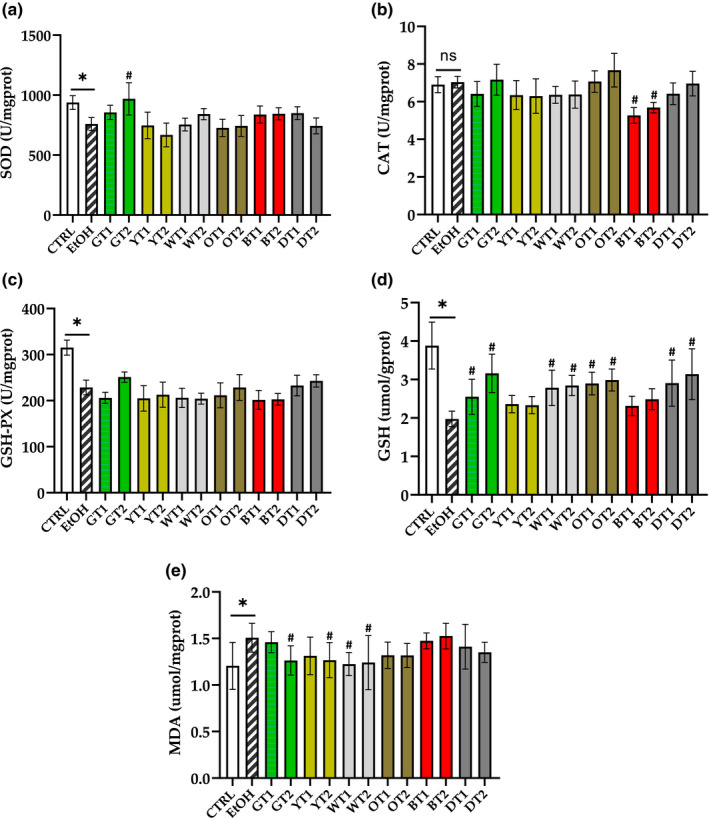
The effects of 12 teas on antioxidant capacity and lipid peroxidation level in mice with fatty liver induced by chronic alcohol consumption. (a) SOD, superoxide dismutase; (b) CAT, catalase; (c) GSH‐Px, glutathione peroxidase; (d) GSH, glutathione; (e) MDA, malondialdehyde. CTRL, the control group; EtOH, the model group; GT1, Selenium‐Enriched Enshi Yulu Tea; GT2, Fried Green Tea; YT1, Yuan'an Luyuan Tea; YT2, Mengding Huangya Tea; WT1, Gongmei White Tea; WT2, White Peony Tea; OT1, Wuyi Narcissus Tea; OT2, Fenghuang Narcissus Tea; BT1, Yihong Tea; BT2, Lapsang Souchong Tea; DT1, Qing Brick Tea; DT2, Pu‐erh Tea. *p < .05, the model group versus the control group; #*p* < .05, the tea extract supplemented group versus the model group

Under normal physiological circumstances, the liver possesses a powerful antioxidant defense system including antioxidant enzymes (SOD, CAT, and GSH‐Px) and non‐enzymatic antioxidants (GSH), which defend against excessive ROS, such as superoxide anion radical and hydrogen peroxide (Dalia et al., 2017). As shown in Figure 6, the sub‐acute intake of alcohol decreased SOD and GSH‐Px activities as well as GSH content and increased the content of MDA in the liver of mice (*p* < .05). These results suggested that sub‐acute excessive alcohol could decline the antioxidant capacity and promote the lipid peroxidation, which exhibited severe hepatocyte injury.

Superoxide dismutase (SOD) and CAT are the essential antioxidant enzymes. The dismutation of two superoxide anions to oxygen and hydrogen peroxide is catalyzed by SOD, and two hydrogen peroxide molecules are degraded into water and oxygen by CAT. Therefore, SOD is also considered as the front line of anti‐free radical enzyme among antioxidant enzymes (Atig et al., 2012). As displayed in Figure 6a, the SOD activity was obviously increased (*p* < .05) by the treatment of Fried Green Tea (GT2) extracts (*p* < .05). It is indicated that Fried Green Tea (GT2) could inhibit the generation of ROS and defend the liver against oxidative stress caused by sub‐acute alcohol consumption. However, the activity of SOD was not elevated in other treatment group compared with the model group. These results indicated that the effects of various teas on SOD were very different. On the other hand, it was reported that the effects of the natural antioxidants on SOD activity were complicated due to the differences in bioavailabilities or antioxidant ingredients (Zhang, Zhou, et al., 2016). The effect of 12 tea extracts on the activity of CAT is provided in Figure 6b. We found that Yihong Tea (BT1) and Lapsang Souchong Tea (BT2) significantly reduced (*p* < .05) the activity of CAT compared with the model group.

GSH‐Px can promote the reaction of hydrogen peroxide and GSH to produce water and glutathione disulfide (GSSG). Moreover, GSH is not only a substrate for GSH‐Px, but also the most important in vivo non‐enzymatic antioxidant, which can eliminate free radicals via its functional groups (Yang et al., 2019). Seen from Figure 6c,d, the supplement of Fried Green Tea (GT2), Fenghuang Narcissus Tea (OT2), Qing Brick Tea (DT1), and Pu‐erh Tea (DT2) enhanced the activity of GSH‐Px (*p* > .05), though there was no statistical significance. On the other hand, the content of GSH in the mice with AFLD was significantly reversed by most of the 12 tea extracts (*p* <.05). Similarly, an elevated tendency in the content of GSH was observed in the group treated with white teas and black teas, although there was no significant difference.

MDA is one of the markers of lipid peroxidation, and the change in the level of MDA can indicate the degree of lipid peroxide and indirectly reflect liver damage (Raza & John, 2007; Wang et al., 2018). Figure 6e presents the effects of 12 tea extracts on the MDA level. We found that four out of 12 tea extract treatment groups obviously reduced the content of MDA when they were compared with the model group (*p* < .05). It is indicated that these teas ameliorated severe lipid peroxidation induced by sub‐acute excessive alcohol consumption.

**FIGURE 7:**
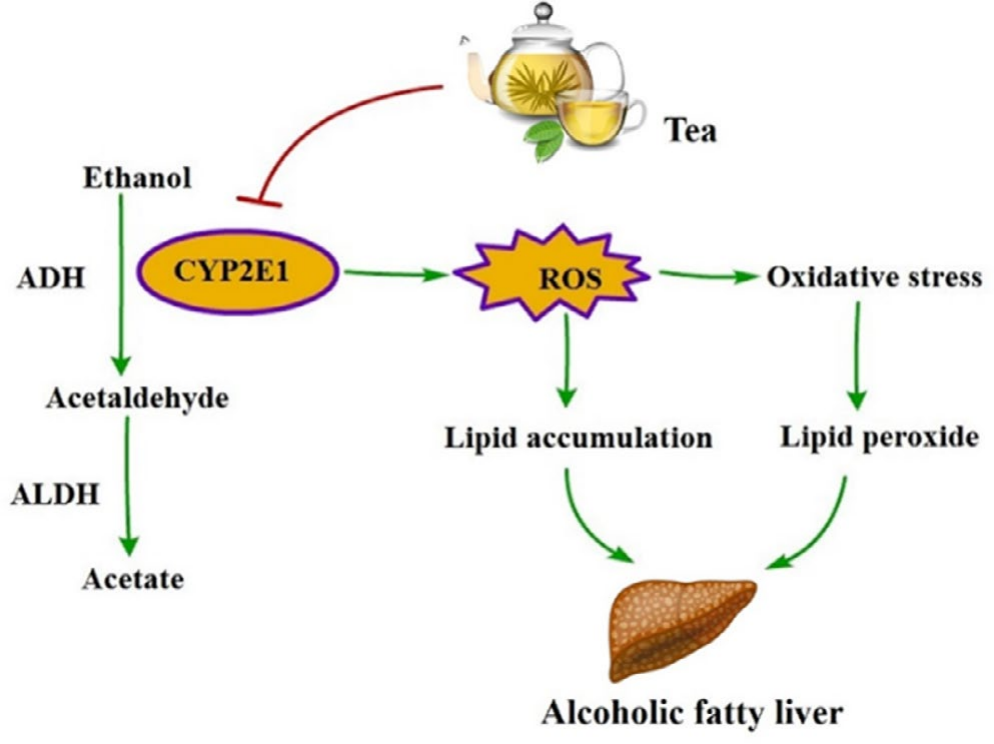
The relationship among tea, ethanol metabolism, and alcoholic fatty liver. Some tea extracts could prevent the formation of alcoholic fatty liver by promoting ethanol metabolism, inhibiting the expression of CYP2E1, ameliorating oxidative stress, and reducing lipid peroxide. ADH, alcohol dehydrogenase; ALDH, acetaldehyde dehydrogenase; CYP2E1, cytochrome P450 2E1; ROS, reactive oxygen species

Increasing studies have demonstrated that tea has an antioxidant property which was mainly attributed to its phenolic compounds, which possess multiple effects like scavenging free radicals and reducing oxidative stress (Lee et al., 2014). In compliance with these studies, our results also suggested that some of the 12 tea extracts could elevate the antioxidant capability and exhibit a significant reduction in lipid peroxidation, which could attenuate oxidative stress damage induced by sub‐acute alcohol consumption (Figure 7), especially Fried Green Tea (GT2).

### 3.7 Phenolic compounds in fried Green Tea, Fenghuang Narcissus Tea, and Pu‐erh Tea

**FIGURE 8:**
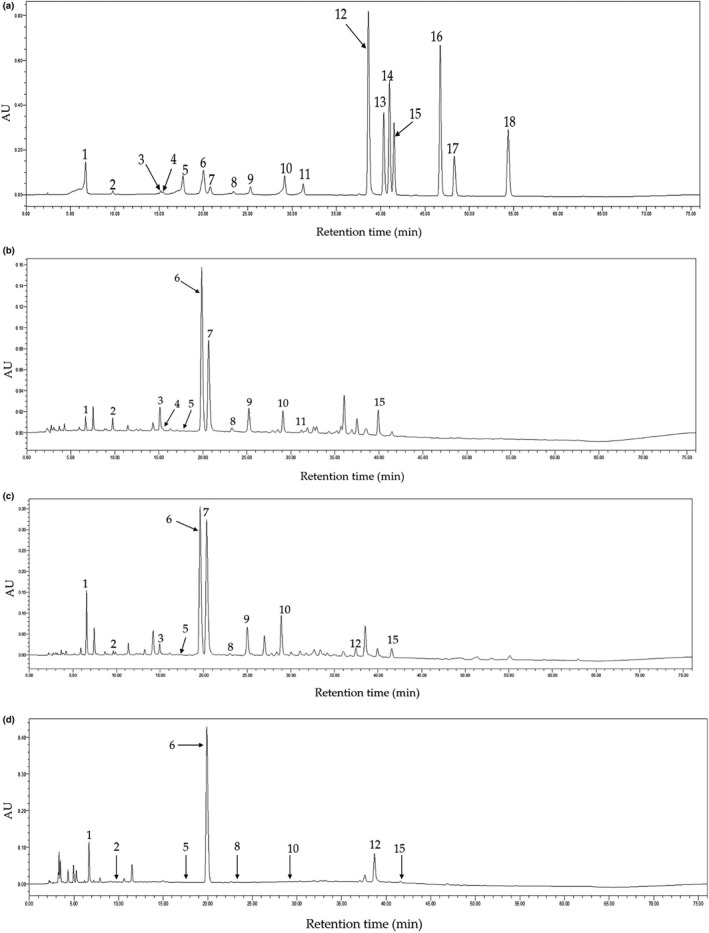
The HPLC chromatograms of the standard compounds (a), Fried Green Tea (b), Fenghuang Narcissus Oolong Tea (c), and Pu‐erh Dark Tea (d) under 254 nm. 1, gallic acid; 2, gallocatechin; 3, epigallocatechin; 4, catechin; 5, chlorogenic acid; 6, caffeine; 7, epigallocatechin gallate; 8, epicatechin; 9, gallocatechin gallate; 10, epicatechin gallate; 11, catechin gallate; 12, ellagic acid; 13, myricetin; 14, quercitrin; 15, astragalin; 16, quercetin; 17, theaflavin; 18, kaempferol

In the present study, the phenolic compounds in Fried Green Tea (GT2), Fenghuang Narcissus Tea (OT2), and Pu‐erh Tea (DT2) extracts were measured by HPLC because they showed stronger inhibitory effects on the hepatic lipid accumulation induced by sub‐acute alcohol consumption. The chromatograms at 254 nm of the standards, Fried Green Tea (GT2), Fenghuang Narcissus Tea (OT2), and Pu‐erh Tea (DT2) are shown in Figure 8. In total, 13 phytochemicals in Fried Green Tea (GT2), Fenghuang Narcissus Tea (OT2), and Pu‐erh Tea (DT2) extracts have been identified and quantified, including gallic acid, gallocatechin(GC), epigallocatechin (EGC), catechin (C), chlorogenic acid, caffeine, epigallocatechin gallate (EGCG), epicatechin (EC), gallocatechin gallate (GCG), epicatechin gallate (ECG), catechin gallate (CG), ellagic acid, and astragalin. However, myricetin, quercitrin, quercetin, theaflavin, and kaempferol have not been determined and quantified in the three teas extracts, which indicated that the concentration of these compounds in the sample solutions was below their detection limits.

According to the results, we found that the detected phenolic substances in Fried Green Tea (GT2), Fenghuang Narcissus Tea (OT2), and Pu‐erh Tea (DT2) extracts were 12, 11, and eight species, respectively. As presented in Figure 8a, the most abundant phytochemicals identified in Fried Green Tea (GT2) were catechins which included eight catechins. Among the eight catechins, EGCG was the most abundant catechin and the content was 56.769 ± 0.199 mg/g DW (Table 5). In addition, the other three types of catechins with higher contents among eight catechins were EGC (19.956 ± 0.083 mg/g DW), GC (14.127 ± 0.129 mg/g DW), and GCG (14.104 ± 0.030 mg/g DW). Besides, Fenghuang Narcissus Tea (OT2) possessed the highest EGCG concentration among three teas, and the value was 76.147 ± 0.125 mg/g DW as indicated in Figure 8b. Moreover, Fenghuang Narcissus Tea (OT2) extracts also included abundant catechins except for CG, while ellagic acid could be detected in it. However, the catechins identified in Pu‐erh Tea (DT2) extracts were only four species with low values, ranging from 0.052 ± 0.004 mg/g DW to 2.339 ± 0.003 mg/g DW. Catechins were the most abundant phytochemicals in Fried Green Tea (GT2) and Fenghuang Narcissus Tea (OT2), while they were much less in Pu‐erh Tea (DT2). It indicates that Fried Green Tea (GT2) possessed the highest in *vivo* antioxidant capacity may be attributed to the most abundant catechins. More and more evidences demonstrated that tea catechins are effective scavengers of reactive oxygen species in *vitro* (Kakutani et al., 2019; Suraphad et al., 2017). Furthermore, it has been reported that the effect of tea and green tea catechins on oxidative stress seems very promising in animal models (Higdon & Frei, 2003).

**TABLE 5:** The contents of main phytochemicals in Fried Green, Fenghuang Narcissus, and Pu‐erh Tea (mg/g DW)

Main phytochemicals	Fried Green Tea	Fenghuang Narcissus Oolong Tea	Pu‐erh Dark Tea
Gallic acid	1.130 ± 0.014	3.914 ± 0.025	2.339 ± 0.003
Gallocatechin	14.127 ± 0.129	3.327 ± 0.010	0.323 ± 0.023
Epigallocatechin	19.956 ± 0.083	7.801 ± 0.150	—
Catechin	5.543 ± 0.272	—	—
Chlorogenic acid	0.781 ± 0.004	0.358 ± 0.001	0.227 ± 0.004
Caffeine	23.274 ± 0.116	19.610 ± 0.131	18.814 ± 0.088
Epigallocatechin gallate	56.769 ± 0.199	76.147 ± 0.125	—
Epicatechin	4.617 ± 0.095	2.008 ± 0.012	0.796 ± 0.002
Gallocatechin gallate	14.104 ± 0.030	14.123 ± 0.012	—
Epicatechin gallate	3.908 ± 0.019	6.317 ± 0.008	0.052 ± 0.004
Catechin gallate	0.819 ± 0.014	—	—
Ellagic acid	—	0.918 ± 0.002	0.734 ± 0.003
Myricetin	—	—	—
Quercitrin	—	—	—
Astragalin	2.036 ± 0.034	0.711 ± 0.003	0.088 ± 0.008
Quercetin	—	—	—
Theaflavin	—	—	—
Kaempferol	—	—	—

Abbreviations: —, means not detected; DW, dry weight of tea.

On the other hand, the three tested tea extracts were found with high contents of caffeine, and the contents varied from 18.814 ± 0.088 to 23.274 ± 0.116 mg/g DW, which might be related to the inhibition of lipid accumulation. It is reported that caffeine could promote lipid metabolism and heat production by converting differentiated 3T3‐L1 adipocytes from white to beige adipocytes (Sugiura et al., 2020).

In conclusion, the most commonly detected ingredients in Fried Green Tea (GT2), Fenghuang Narcissus Tea (OT2), and Pu‐erh Tea (DT2) extracts were catechins and caffeine, which could contribute to the effects of in vivo antioxidant activity and the inhibition of lipid accumulation.

Generally, tea is classified into six categories according to fermentation degree, including green tea, yellow tea, white tea, oolong tea, black tea, and dark tea. Tea contains abundant phytochemicals, but the bioavailability of tea phytochemicals is relatively low (Tang et al., 2019). Tea has shown many health functions, such as antioxidant, anti‐inflammatory, immuno‐regulatory, cardiovascular‐protective, hepatoprotective, anti‐obesity, anti‐diabetic, and anticancer effects (Cao et al., 2019; Shang et al., 2021; Tang et al., 2019). Green tea is usually drunk in the East, and black tea is favorable in West. Tea is generally safe for drinking with less than 12 gram every day, although tea could contain little heavy metals, pesticide residues and mycotoxins from plantation, manufacture, and storage (Shang et al., 2021; Tang et al., 2019).

## 4 CONCLUSIONS

In this study, the effects of 12 selected teas on AFLD were evaluated and compared. Based on the histological evaluation and the determination of serum and hepatic TG concentrations, the results revealed that these teas exhibited different inhibitory effects on AFLD. The preventive effect of the teas on AFLD was associated with the inhibition of CYP2E1 expression, the promotion of alcohol metabolism, the depression of AST and ALT activities, the elevation of in vivo antioxidant capacity, and the decrease of lipid peroxidation. Among these teas, Fried Green Tea (GT2), Fenghuang Narcissus Tea (OT2), and Pu‐erh Tea (DT2) showed stronger inhibitory effects on the hepatic lipid accumulation induced by sub‐acute alcohol consumption. Therefore, these teas have great potential to be developed into functional foods for preventing AFLD and are suggested to drink accompanied with alcohol consumption. Furthermore, the most commonly detected ingredients in Fried Green Tea (GT2), Fenghuang Narcissus Tea (OT2), and Pu‐erh Tea (DT2) extracts were catechins and caffeine, which could contribute to the effects of antioxidant capacity and the inhibition of lipid accumulation. This study also provides the public and nutritionists with new information regarding the effects of different teas against AFLD.

## ETHICAL APPROVAL

The authors declare no conflict of interest. The study was conducted according to the guidelines of the Declaration of Helsinki. The study's protocols and procedures were ethically reviewed and approved by the Animal Care Committee at the School of Public Health, Sun Yat‐Sen University (approval number: 2019‐002; 28 February 2019).

## Data Availability

The data are kept in School of Public Health, Sun Yat‐Sen University.
